# Antimicrobial prescription patterns in East Africa: a systematic review

**DOI:** 10.1186/s13643-022-02152-7

**Published:** 2023-02-14

**Authors:** Joan Acam, Paul Kuodi, Girmay Medhin, Eyasu Makonnen

**Affiliations:** 1 Center for Innovative Drug Development and Therapeutic Trials for Africa (CDT-Africa), College of Health Sciences, Addis Ababa University, P.O. Box 9086, Addis Ababa, Ethiopia; 2 Pope John’s Hospital — Aber, Lira Municipality, Uganda; 3 Azrieli Faculty of Medicine, Bar-Ilan University, Safed, Israel

## Abstract

**Background:**

Antimicrobial resistance is currently a recognized global health problem stemming from poor antibiotic stewardship by health workers and inappropriate antimicrobial use by patients. Data showing the extent of poor antimicrobial stewardship in low- and middle-income countries are scanty though high incidences of antimicrobial resistance are increasingly reported in many settings across the globe. The objective of the present study was, therefore, to evaluate prescriptions for antimicrobials in East Africa.

**Methods:**

A comprehensive literature search strategy that includes text words and medical subject headings was developed and applied to predefined electronic databases. Two authors independently screened the titles and abstracts of the outputs of the literature search. Full texts were then independently reviewed by the first and the second authors. Eligible studies were formally assessed for quality and risk of bias using a scoring tool. Extracted data from included studies were combined in a meta-analysis where appropriate and presented using forest plots and tables or in a narrative text. Where data were available, subgroup analyses were performed.

**Results:**

A total of 4284 articles were retrieved, but only 26 articles were included in the review. The majority of the included studies (30.8%) were retrieved from Ethiopia, followed by Sudan, Kenya, and Tanzania each contributing 19.2% of the included studies. The overall proportion of encounters with antimicrobials reported by the included studies was 57% CI [42–73%]. Ethiopia had an overall patient encounter with antimicrobials of 63% [50–76%] followed by Sudan with an overall encounter with antimicrobials of 62% CI [34–85%]. Included studies from Kenya reported an overall encounter with antimicrobials of 54% CI [15–90%], whereas included studies from Tanzania reported an overall patient encounter with antimicrobials of 40% CI [21–60%].

**Conclusion:**

Prescription patterns demonstrated in this review significantly deviate from WHO recommendations suggesting inappropriate antimicrobial use in the East African countries. Further studies have to be pursued to generate more information on antimicrobial use in this region.

**Supplementary Information:**

The online version contains supplementary material available at 10.1186/s13643-022-02152-7.

## Introduction

Antimicrobial resistance is currently a recognized global health problem stemming from poor antibiotic stewardship by health workers and improper use of antimicrobial by patients [1]. Data showing the extent of poor antimicrobial stewardship in low- and middle-income countries is scanty though high incidences of antimicrobial resistance are increasingly reported in many settings across the globe [2]. Reports indicate that misuse of antimicrobials including overprescription and prescription without proper identification of offending pathogens in humans and animals is some of the main drivers of the currently witnessed antimicrobial resistance [3].

Studies revealed a more than 65% dramatic increase in antibiotic consumption between 2000 and 2015 fuelled by excessive antibiotic prescription in low- and middle-income countries [4]. One study reported the increasing trends of antimicrobial resistance due to the COVID-19 pandemic resulting from irrational antimicrobial treatment [5]. It is estimated that 10 million deaths will occur in Africa and Asia by 2050 if improper antimicrobial use is not tackled as a matter of emergency [5]. Several factors have attributed to the rise in antimicrobial use especially in Africa and other low- and middle-income countries: high burden of infectious diseases, poor antibiotic stewardship due to inadequate training of health professionals, lack of essential diagnostic equipment, widespread over-the-counter (OTC) sale of antibiotics, and weak antibiotic regulatory environment [6, 7].

Literature reporting on antibiotic use and prescription patterns is available in a lot of small studies with scanty synthesized evidence for Africa and other low- and middle-income countries [8, 9]. Yet devising interventions to combat the current global upsurge of antimicrobial resistance requires guidance from quality evidence whose availability is limited. This study is aimed to synthesize available data on this topic to avail policy relevant evidence on antimicrobial prescriptions in East Africa in order to guide decisions on antimicrobial resistance interventions.

### Rationale for the review

Institution of antibiotics stewardship interventions as currently recommended by WHO requires guidance from quality evidence-based data. Unfortunately, such data are scanty for many low- and middle-income countries. The rationale for this review was therefore to map using systematic review and meta-analysis methods, the prescription patterns, and to determine the antimicrobial level of appropriate use in East African states by synthesizing available evidence from East African countries.

### Objectives

#### Primary objectives

The primary objectives of this study included the following:

- To characterize the antimicrobial prescription patterns in East Africa
- To determine the proportion of patient encounters with antimicrobial prescriptions in East Africa

#### Secondary objective

- To determine the magnitude of inappropriate antimicrobial use in Africa

## Methods

### The literature search strategy employed

The literature search strategy used both text words and medical subject heading (MeSH) terms (all fields). Key terms such as “antimicrobial” or “antibiotic” or “anti-infective agent” were used to develop a search strategy. No time and language restrictions were applied during database searches.

### Example of the search strategy developed and on PubMed database

(("primary health care"[mesh] OR primary care[tw] OR primary health*[tw] OR community health*[tw] OR community care[tw] OR community worker*[tw] OR clinic[tw] OR clinics[tw] OR “general practitioners” [mesh] OR general practi*[tw] OR family medicine[tw] OR family practi*[tw] OR “physicians, family” [mesh] OR family physician*[tw] OR family doctor*[tw] OR "physicians, primary care"[mesh])) AND (("anti-bacterial agents"[Pharmacological Action] OR "anti-bacterial agents"[MeSH Terms] OR "anti-infective agents"[Pharmacological Action] OR "anti-infective agents"[MeSH Terms] OR antibiotic*[tw] OR antimicrobial*[tw] OR antibacterial*[tw] OR anti bacterial*[tw] OR anti-infective*[tw])) AND ("therapeutic use"[sh] OR "drug prescriptions"[mesh] OR "drug utilization"[mesh] OR “inappropriate prescribing” [mesh] OR "drug utilization review" [mesh] OR "practice patterns, physicians'"[mesh] OR use[tiab] OR user*[tiab] OR used[tiab] OR overuse*[tiab] OR underuse*[tiab] OR misuse*[tiab] OR utiliz*[tiab] OR overutili*[tiab] OR underutili*[tiab] OR prescri*[tw] OR overprescri*[tiab] OR underprescri*[tiab])

### Types of studies included in the review

We included in this review studies conducted in East Africa that reported the proportion of patients receiving any antibiotic prescription irrespective of facility setting or level. The following study types were included in the review: cross-sectional studies, cohort studies, and RCTs (randomized controlled trials).

Reviews of all kinds, economic evaluation studies, qualitative studies, mathematical modeling, and non-primary research publications such as commentaries, editorials, and conference proceedings were excluded. Studies reporting antibiotic use in animals, i.e., those focused on veterinary use of antibiotics and those focused on special cohorts of patients such as surgical prophylaxis where the use of antibiotics is justified, were excluded.

### Data collection and analysis

#### Selection of studies

All electronic database outputs were imported into Rayyan software for screening and selection. The first and second authors independently screened 100% titles and abstracts for inclusion of potentially eligible studies sourced from the database searches. Titles and abstracts in non-English languages were translated into English using Google Translate. The first reviewer (J. A.) collected full-text articles/publications of potentially eligible studies, and then J. A. and the second reviewer (PK) independently screened 100% of full-text articles for inclusion. Where disagreement occurred between the two reviewers, the last author (EM) was consulted. Each step of the study selection process was documented, and where a study was excluded, the reason(s) for exclusion was recorded and entered into the PRISMA flow diagram.

### Data extraction and management

Data were independently extracted in text, tables, and figures of the included studies by the first and second authors and recorded on a standardized, pre-designed extraction form designed in Excel. In the case of unclear data, reviewers contacted corresponding authors listed in the articles for clarifications. Data management was the duty of the first author (J. A.) in consultation with the second author (P. K.). Completed data extraction forms were maintained on both a password-secured laptop and USB memory stick and exported to STATA for analysis.

The following data points were extracted from the included studies:

- Study characteristics: Year(s) of data collection, study design, source of data, population or participants, and objectives of the study
- Study setting: Country, income level, and health facility level
- Outcome measures: Number of individuals receiving at least one antimicrobial prescription to the number of persons attending a given health facility within a specified period

### Risk-of-bias assessment

The methodological quality of studies including the risk of bias was assessed using a checklist to assess for internal and external validity. A modified checklist originally developed by Hoy and colleagues was used to score; sampling strategies used, outcome assessment, outcome measurement, and statistical reporting and higher overall scores represented higher methodological quality [10]. Each article was independently scored by the first and second authors in consultation with the last author, attached as supplemental file, S1.

### Treatment of missing data

Authors of articles with missing data were contacted to provide the missing data points. In cases where the missing data were impossible to obtain, full descriptions are provided about the nature of the missing data and the implications on the results in the reporting of this systematic review.

### Assessment of heterogeneity

Forest plots were used to assess the presence of statistical heterogeneity. We assessed heterogeneity by calculating *χ*^2^ (threshold *P* > 0.1) and *I*^2^ statistics (threshold *I*^2^ > 40%). The values of *I*^2^ were categorized for heterogeneity as follows: “not important” (0 to 40%), “moderate” (41 to 60%), “substantial” (61 to 80%), and “considerable” (81 to 100%). Where “not important” or “moderate” heterogeneity exists between studies (*I*^2^≤ 40%), the outcomes were pooled in a meta-analysis and reported using forest plots. Where “substantial” or “considerable” heterogeneity exists between studies (*I*^2^ > 40%), the outcomes were pooled and reported in narrative form using forest plots.

### Data synthesis

Data from the included studies were combined using a random-effects model to account for variability between studies. This is because substantial between-studies heterogeneity was anticipated considering the different study designs included in the systematic review. STATA software (College Station, Texas 77845, USA) was used to perform the meta-analysis.

### Sensitivity analysis

Sensitivity analyses were performed to assess if methodological differences in outcome measurement influenced the review results.

## Results

### Included studies

The literature search retrieved 4284 records from 8 databases searched. From the PubMed database, 224 records were retrieved. Results from other databases searched include the following: EBSCOhost, 1018 records, Web of Science, 458 records, Cochrane Library, 1118 records, Scopus, 1452 records, International Clinical Trials Registry Platform (ICTRP), 11 records, and from MedNar, 3 records were retrieved.

After removal of 1224 duplicates, 3060 records were subjected to a title and abstract screening. During the title and abstract screening process, 3010 studies were excluded for failure to meet the inclusion criteria. The excluded studies included 204 review articles, 905 nonintervention studies, 1161 non-primary research studies, and 70 records excluded for other reasons.

**Fig. 1:**
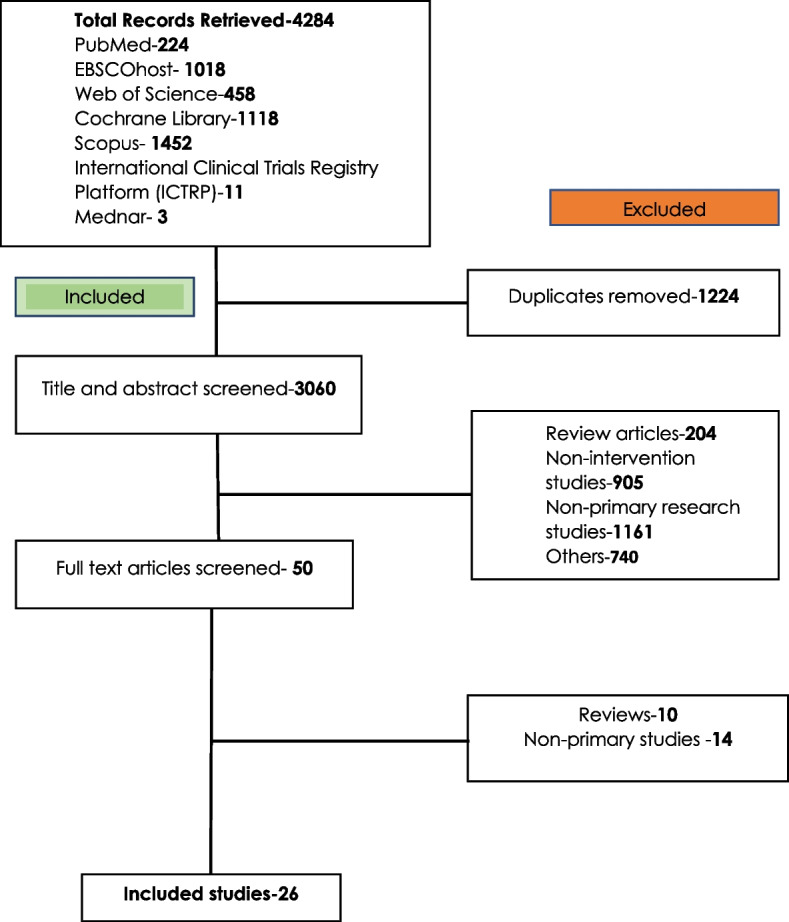
Flow diagram showing process of selection of eligible articles [11–14]

Fifty studies were subjected to a full-text screening. After full-text screening, 26 six studies met the inclusion criteria and were thus selected as the final studies for inclusion in the systematic review. Figure 1 shows the process followed to arrive at the final studies included in the review.

### Patterns of antimicrobial prescriptions in East Africa

The patterns of antimicrobial prescriptions assessed in this systematic review followed the recommended WHO metrics for assessing prescriptions of drugs which include the proportion of patient encounters with antimicrobials prescriptions, the proportion of patient encounters with injectable antimicrobials prescriptions, the proportion of patient encounters with antimicrobial prescriptions from the essential medicines list, and the proportion of encounters with antimicrobial prescriptions in generic names.

#### Proportions of patient encounters with antimicrobials prescriptions in East African states

**Fig. 2:**
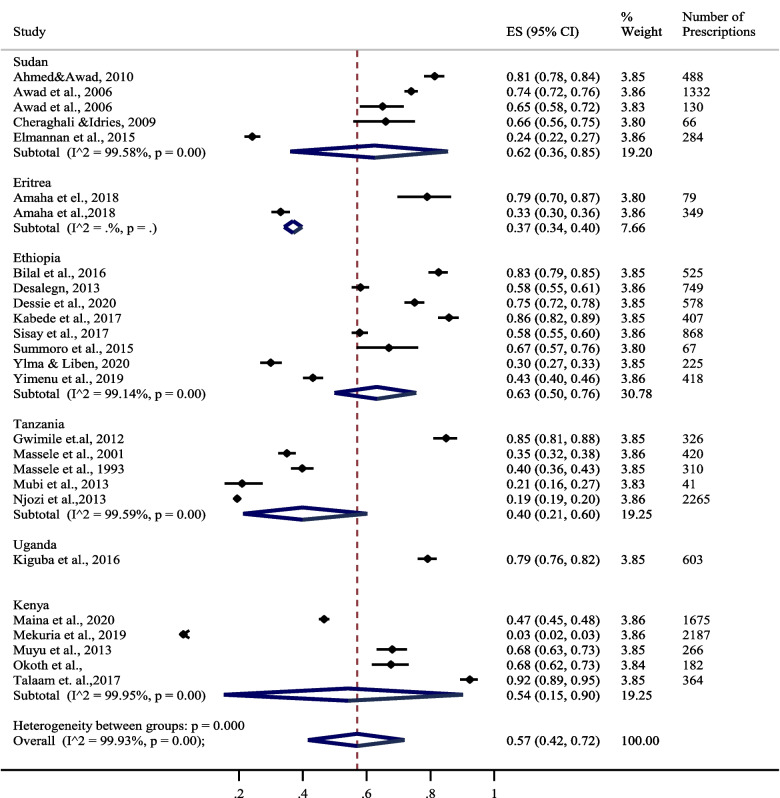
Forest plot showing proportions of patient encounters with antimicrobials in East African states [15–36]

The overall proportion of encounters with antimicrobial reported by the included studies was 57% CI [42–73%]. Ethiopia had an overall patient encounter with antimicrobials of 63% [50–76%] followed by Sudan with an overall encounter with antimicrobials of 62% CI [34–85%]. Included studies from Kenya reported an overall encounter with antimicrobials of 54% CI [15–90%], whereas included studies from Tanzania reported an overall patient encounter with antimicrobials of 40% CI [21–60%]. Included studies from Uganda and Eritrea reported patient encounters with antimicrobials of 79% CI [76–82%] and 37% CI [34–40%], respectively. Figure 2 shows the proportion of patient encounters with antimicrobial prescriptions in East African states.

#### Proportion of patient encounters with injectable antimicrobials prescriptions in East African states

**Fig. 3:**
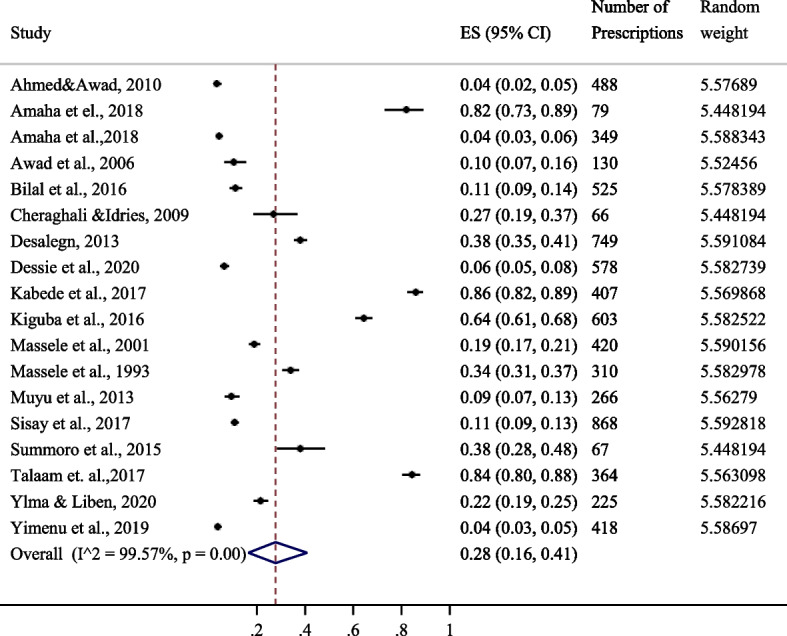
Proportion of encounters with injectable antimicrobials in East Africa [15–23, 25, 26, 30, 32, 35, 36]

Eighteen studies reported patient encounters with injectable antimicrobials. Overall, the patient encounter with injectable antimicrobials was 28% CI [16–41%] for all the East African states. Heterogeneity among the included studies in the meta-analysis was 99.6%, *p*-value of 0.000. The forest plot in Fig. 3 shows a summary of studies reporting proportions of patient encounters with injectable antimicrobial agents.

#### Proportion of patient encounters with antimicrobial prescriptions from the essential medicines list

**Fig. 4:**
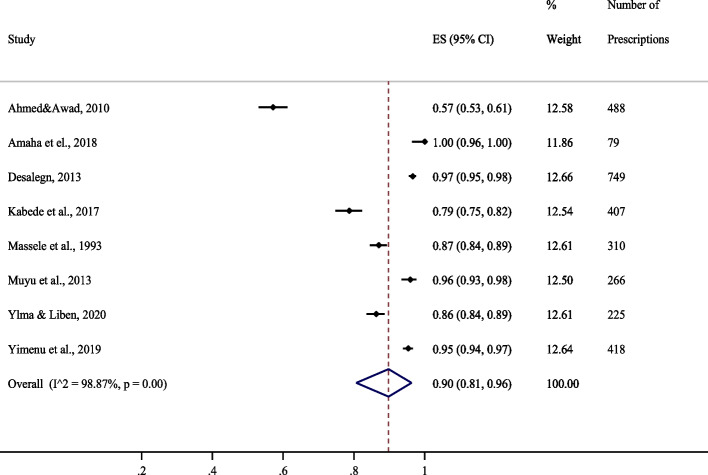
Proportion of encounters with antimicrobial prescriptions from essential medicines list [18–21, 25, 30, 35]

Eight out of the 26 studies that met the review’s inclusion criteria reported patient encounters with antimicrobial prescriptions from the essential medicines list. Overall, the proportion of prescriptions from the essential medicines list was 90% CI [81–96%]. Figure 4 summarizes the results from studies reporting antimicrobial prescriptions from the essential medicines list.

#### Proportion of encounters with antimicrobial prescriptions in generic names

**Fig. 5:**
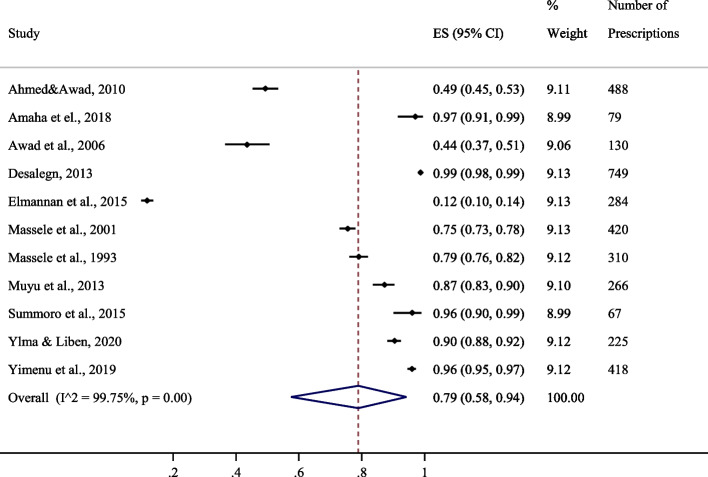
Proportion of encounters with antimicrobial prescriptions in generic names [16, 19–21, 23–25, 30, 32, 35]

Eleven studies reported patient encounters with antimicrobial prescriptions in generic names. Overall, prescriptions in generic names were 79% CI [58–94%]. Figure 5 provides a summary of the proportions of patient encounters with antimicrobial prescriptions in East Africa.

#### Appropriateness of antimicrobial prescriptions in East Africa

To assess the appropriateness of antimicrobial prescriptions in East Africa, systematic review findings were compared with WHO recommended values. The systematic review findings and the WHO recommended values are summarized in Table 1.

**Table 1:** Comparison of systematic review findings on patterns of antimicrobial use and WHO recommended ideal values

Patterns of drug use (including antimicrobials)	Systematic review finding	WHO recommendation
% encounters with antimicrobials	57% (42–72%)	20% or less
% encounter with injection prescriptions	28% (16–41%)	10% or less
% generic name prescribing	79% (58–94%)	100%
% drugs prescribed from EML	90% (81–96%)	100%

## Discussion

According to available literature, we found the patterns of antimicrobial prescriptions across East Africa countries to be heterogenous. Majority of the studies (30.8%) included in this review originated from Ethiopia, followed by Sudan, Kenya, and Tanzania each contributing 19.2% of the included studies. In addition, Ethiopia, Sudan, Kenya, and Uganda had patient encounters with antimicrobial prescriptions greater than 50%. The high frequency of patient encounter with antimicrobial agents could be due to the clinicians practice of prescribing antimicrobial agents without culture and sensitivity tests, symptomatic management, empirical treatment, inadequate use of available country-specific treatment guidelines, and insufficient knowledge and level of experience of the prescribers [37, 38]. Furthermore, none of the countries represented in the systematic review reported lower antimicrobial prescription encounters than the WHO recommended value of 20% or less.

The least number of included studies was retrieved from Uganda (3.9%). This review showed that East African countries were not at the same level regarding the research-based evidence of antimicrobial resistance patterns. The results from this review also demonstrates how much research is being carried in each country represented in the review regarding antimicrobial use patterns. Considering the global threat posed by antimicrobial resistance, perhaps countries with few research being carried out on use patterns and antimicrobial resistance should focus more on this research agenda as a matter of public health priority. Studies on antimicrobial use audits and implementation of interventions should be done in order to combat this global emergency that has far-reaching health implications on the economy and health of the population in these countries.

Overall, patient encounter with antimicrobial agents in East Africa was 57%. This percentage of antimicrobial encounter is higher than the WHO recommended value of 20% or less [39]. Furthermore, none of the countries represented in the systematic review reported lower antimicrobial prescription encounters less than the WHO recommended value of 20% or less. Several countries from East Africa were included with the hope to make meaningful comparison.

The overall patient encounter with injectable antimicrobials prescriptions was found to be 28%. This is also a deviation from the ideal WHO recommended value of 10% or less for injectable prescriptions [39]. Prescription of antimicrobials by generic names and prescriptions of antimicrobials from the essential medicines list were found to be lower than the recommended value of 100%. Countries in LMIC including East African countries have limited antimicrobial drugs in their essential drugs list. The increased inappropriate antimicrobial use in low-income countries might be attributed to inappropriate drug policies, poor health systems, a few skilled workforce, and few diagnostics coupled with few of antimicrobial agents listed in their essential drug lists.

In most East African countries considered in this review, there is a high level of pharmaceutical marketing and promotion of brand names by pharmaceutical companies with little regulation by the government [40]. Due to the extensive promotions, both prescribers and the patients have over time developed more confidence in the brand names which they are familiar with [41, 42].

### Strengths and limitations of the review

A comprehensive literature search strategy was used to retrieve studies for inclusion in the review. A high level of heterogeneity made a meta-analysis not credible; therefore, results were synthesized narratively, and the forest plots were used for illustrative purposes only and to augment the narrative synthesis.

## Conclusion and recommendations

Prescription patterns have been demonstrated in this review to deviate significantly from WHO recommendations suggesting inappropriate antimicrobial use leading to antimicrobial resistance. Because of the high-level heterogeneity among included studies in this systematic review, the true extent of the poor antimicrobial stewardship in East Africa needs further evaluation using well-designed and well-conducted clinical trials. The findings of this study highlight the areas for action to improve antimicrobial prescription practices.

## Supplementary Information

**Additional file 1.** Risk of Bias Assessment.

**Additional file 2.** Search strategy for use in EBSCOhost, Web of Science, Cochrane Library, Scopus, International Clinical Trials Registry Platform (ICTRP) and Mednar databases.

**Additional file 3.** PROTOCOL Systematic review.

## Data Availability

All data generated during the review of included studies are reported in this published article and the supplementary information files.

## Abbreviations

OTC: Over the counter
CENTRAL: Cochrane Central Register of Controlled Trials
CINAHL: Cumulative Index to Nursing and Allied Health Literature
ICTRP: International Clinical Trials Registry Platform
PRISMA: Preferred Reporting Items for Systematic Reviews and Meta-Analyses
RCT: Randomized controlled trial
