# Supplementary material for: Antimicrobial prescription patterns in East Africa: a systematic review
Source: Syst Rev. 2023 Feb 14;12:18. doi: 10.1186/s13643-022-02152-7 (PMC9927054; doi:10.1186/s13643-022-02152-7)
Supplement: Supplementary file 2 — Additional file 2. Search strategy for use in EBSCOhost, Web of Science, Cochrane Library, Scopus, International Clinical Trials Registry Platform (ICTRP) and Mednar databases. [file 13643_2022_2152_MOESM2_ESM.docx]

| **Additional file 2: Search strategy for use in EBSCOhost, Web of Science, Cochrane Library, Scopus, International Clinical Trials Registry Platform (ICTRP) and Mednar databases** | | |
| --- | --- | --- |
| **Query** | **Fields** | **Search term** |
| #1 | All | (((((antimicrobial) OR (antiviral)) OR (antimalarial)) OR (antifungal)) OR (antibiotic)) |
| #2 | All | ((((prescription) OR (patterns)) |
| #3 | All | (East Africa)) |
| #4 | #1 AND #2 AND #3 | |
