# Supplementary material for: Antimicrobial prescription patterns in East Africa: a systematic review
Source: Syst Rev. 2023 Feb 14;12:18. doi: 10.1186/s13643-022-02152-7 (PMC9927054; doi:10.1186/s13643-022-02152-7)
Supplement: Supplementary file 3 — Additional file 3. PROTOCOL Systematic review. [file 13643_2022_2152_MOESM3_ESM.docx]

**PROTOCOL**

# **A systematic review and meta-analysis of antimicrobial prescriptions in Africa.**

Joan Acam^1^ , Paul Kuodi Otiku^2^

1

2 Faculty of Health Sciences, Department of Public Health, Lira University

**Abstract**

**Background**

Antimicrobial resistance is currently a recognised global health problem stemming from poor antibiotic stewardship by health workers among other factors. Quality data representative of the extent of poor antimicrobial stewardship in low- and middle-income countries is scanty, but high incidences of antimicrobial resistance are increasingly being reported in many settings across the globe.

**Methods**

A comprehensive literature search strategy that includes text words and medical subject headings will be developed and applied to predefined electronic databases. Two authors will independently screen the titles and abstracts of the output of the literature search. Full texts will then be independently reviewed by the first author and the second authors. Eligible studies will be formally assessed for quality and risk of bias using a scoring tool. Extracted data from included studies will be combined in a meta-analysis where appropriate and presented using forest plots and other graphics or in a narrative text. Where data is available, sub-group analyses will be performed.

**Ethics and dissemination**

The systematic review and meta-analysis will use data extracted from publicly available research articles and does not involve human or animal research participants. The study therefore poses no risks and is exempt from ethical review.

**Systematic review registration**

This protocol is registered with the International Prospective Register of Systematic Reviews (PROSPERO), registration number-……………………………… and publicly accessible at: ……………………………………………………

**Strengths and limitations**

The protocol will use a comprehensive literature search strategy. Limited reporting and poorly conducted studies can affect the achievement of study objectives.

**Introduction**

Antimicrobial resistance is currently a recognised global health problem stemming from poor antibiotic stewardship by health workers among other factors [1]. Quality data representative of the extent of poor antimicrobial stewardship in low- and middle-income countries is scanty, but high incidences of antimicrobial resistance are increasingly being reported in many settings across the globe [2]. Reports indicates that misuse of antimicrobials including over prescription and prescription without proper identification of offending pathogens in humans and animals are some of the main drivers of the currently witnessed antibiotic resistance [3].

A recent study to estimate the global consumption of antibiotics revealed a more than 65% dramatic increase in antibiotic consumption between 2000 and 2015 fuelled by excessive antibiotic prescription in low- and middle-income countries [4]. Several factors have been attributed to the rise in antimicrobial use especially in Africa and other low- and middle-income countries: high burden of infectious diseases, poor antibiotic stewardship due to inadequate training of health professionals, lack of essential diagnostic equipment, widespread over the counter (OTC) sale of antibiotics, and weak antibiotic regulatory environment [5, 6].

Literature reporting on antibiotic use and prescription patterns is scanty for Africa and other low- and middle-income countries [7, 8]. Yet the institution of interventions to combat the current global upsurge of antimicrobial resistance requires guidance from quality evidence. This study is aimed at synthesizing available data on this topic to avail evidence on antibiotic prescriptions in Africa in order to guide decisions on antimicrobial resistance interventions.

**Rationale for the review**

Institution of antibiotics stewardship interventions as currently recommended by WHO [9] requires guidance from quality evidenced based data. Unfortunately, such data is scanty for many low-and middle-income countries. The rationale for this review is therefore to map using systematic review and meta-analysis methods, the prescription patterns and antimicrobial stewardships in Africa by synthesising available evidence from African countries.

**Methods**

**Objectives**

**Primary objective(s):**

**The primary objectives of this study include:**

- To characterise the antibiotic prescription patterns in Africa
- To determine the proportion of patients receiving any antibiotic prescription in Africa

**Secondary objective(s):**

- To determine the magnitude of inappropriate antimicrobial use in Africa

**Search strategy to be employed**

The literature search strategy will use both text words and medical subject heading (MeSH) terms (all fields). Key terms such as; “antimicrobial” or “antibiotic” or “anti-infective agent” will be used to develop a search strategy. Filters will be applied to access studies published from 1^st^ January 2010 through to the present date and without application of any language restriction.

**Example of search strategy developed for use on PubMed database**

(("primary health care"[mesh] OR primary care[tw] OR primary health*[tw] OR community health*[tw] OR community care[tw] OR community worker*[tw] OR clinic[tw] OR clinics[tw] OR “general practitioners”[mesh] OR general practi*[tw] OR family medicine[tw] OR family practi*[tw] OR “physicians, family”[mesh] OR family physician*[tw] OR family doctor*[tw] OR "physicians, primary care"[mesh]))

AND

(("anti-bacterial agents"[Pharmacological Action] OR "anti-bacterial agents"[MeSH Terms] OR "anti-infective agents"[Pharmacological Action] OR "anti-infective agents"[MeSH Terms] OR antibiotic*[tw] OR antimicrobial*[tw] OR antibacterial*[tw] OR anti bacterial*[tw] OR anti-infective*[tw]))

AND

("therapeutic use"[sh] OR "drug prescriptions"[mesh] OR "drug utilization"[mesh] OR “inappropriate prescribing”[mesh] OR “drug utilization review”[mesh] OR "practice patterns, physicians'"[mesh] OR use[tiab] OR user*[tiab] OR used[tiab] OR overuse*[tiab] OR underuse*[tiab] OR misuse*[tiab] OR utiliz*[tiab] OR overutili*[tiab] OR underutili*[tiab] OR prescri*[tw] OR overprescri*[tiab] OR underprescri*[tiab])

**Types of studies to be included in the review**

We will include in this review studies conducted in Africa that reports the proportion of patients receiving any antibiotic prescription irrespective of facility setting/level. The following study types will be included in the review: cross-sectional studies, cohort studies, and RCTs (randomized controlled trials) and non-RCTRs.

Reviews of all kinds, economic evaluation studies, qualitative studies, mathematical modelling and non primary study publications such as: commentaries, editorials and conference proceedings will be excluded. Studies reporting antibiotic use in animals i.e. those focused on veterinary use of antibiotics, and those focused on special cohorts where use of antibiotics is justified, will also be excluded.

**Data collection and Analysis**

**Selection of studies**

All electronic database outputs will be imported to Rayyan Software for screening and selection

[10]. The first and second author will independently screen 100% titles and abstracts for inclusion of potentially eligible studies sourced from database searches. Titles and abstracts in non-English languages will be translated into English using Google Translate. JA will collect full-text articles/publications of potentially eligible studies and then JA and PO will independently screen 100% of full-text articles for inclusion. Where disagreement may occur between the two authors, the last author () will be consulted. We will record the selection process with reasons for exclusion using a PRISMA flow diagram.

**Data extraction and management**

Data will be independently extracted in text, tables and figures of the included studies by the first and second authors and recorded on a standardised, pre-designed extraction form. In the case of unclear data, we will contact corresponding authors to clarify. Data management will be the duty of the first author in consultation with the second author. Completed data extraction forms will maintained on both a password secured laptop and USB memory stick and exported to STATA for analysis.

The following data points will be extracted from included studies:

Study characteristics: year(s) of data collection, study design, source of data, population of participants and objectives of the study.

Study setting: country, income level, health facility level.

Outcome measures: number of individuals receiving at least one antibiotic prescription to the number of persons attending a given outpatient clinic within a specified time period.

**Risk of bias assessment**

The methodological quality of studies including risk of bias will be assessed using a checklist to assess for internal and external validity. A modified check list originally developed by Hoy and colleagues[11] will be used to score; sampling strategies used, outcome assessment, outcome measurement and statistical reporting and higher overall scores will represent higher methodological quality. Each article will be independently scored by the first and second author in consultation with the last author.

**Treatment of missing data**

Authors of articles with missing data will be contacted to provide the missing data points. In cases where the missing data will be impossible to obtain, a full description will be provided about the nature of the missing data and the implications on the results in the reporting of this review.

**Assessment of Heterogeneity**

We will use forest plots to assess the presence of statistical heterogeneity. We will assess heterogeneity by calculating Chi^2^ (threshold P > 0.1) and I^2^ statistics (threshold I^2^ > 40%). The values of I^2^ will be categorized for heterogeneity as follow: “not important” (0 to 40%), “moderate” (41 to 60%) and “substantial” (61 to 80%) and “considerable” (81 to 100%). Where “not important” or “moderate” heterogeneity exists between studies (I^2^ $\leq$ 40%), the outcomes will be pooled in a meta-analysis and reported using forest plots. Where “moderate” or “substantial” heterogeneity exists between studies (I^2^ > 40%), the outcomes will be pooled and reported in narrative form using forest plots.

**Assessment of reporting bias**

We will use funnel plots to assess the presence of reporting bias using the Egger test.

**Data synthesis**

Data from the included studies will be combined using random effects model to account for variability between studies this is because substantial between-studies heterogeneity is anticipated. STATA software (College Station, Texas 77845 USA) will be used to perform the meta-analysis.

**Sensitivity analysis**

Sensitivity analyses will be performed to assess if methodological differences in outcome measurement influenced the review results.

**Discussion**

Available literature reports high rates of antimicrobial prescription in Africa and low- and middle-income countries with a subset of studies reporting poor antibiotic stewardship. The true extent of the reported poor antimicrobial stewardship needs further evaluation. The findings of this study will highlight the areas for action to improve prescription practices.

**References**

1. Holmes, A.H., et al., *Understanding the mechanisms and drivers of antimicrobial resistance.* The Lancet, 2016. **387**(10014): p. 176-187.

2. Organization, W.H., *Antimicrobial resistance: global report on surveillance*. 2014: World Health Organization.

3. Laxminarayan, R., et al., *Antibiotic resistance—the need for global solutions.* 2013. **13**(12): p. 1057-1098.

4. Klein, E.Y., et al., *Global increase and geographic convergence in antibiotic consumption between 2000 and 2015.* 2018. **115**(15): p. E3463-E3470.

5. Organization, W.H., *Antimicrobial resistance and primary health care*. 2018, World Health Organization.

6. Auta, A., et al., *Global access to antibiotics without prescription in community pharmacies: A systematic review and meta-analysis.* 2019. **78**(1): p. 8-18.

7. Versporten, A., et al., *Antimicrobial consumption and resistance in adult hospital inpatients in 53 countries: results of an internet-based global point prevalence survey.* 2018. **6**(6): p. e619-e629.

8. Kardas, P., et al., *A systematic review and meta-analysis of misuse of antibiotic therapies in the community.* 2005. **26**(2): p. 106-113.

9. Organization, W.H., *World health statistics 2015*. 2015: World Health Organization.

10. Ouzzani, M., et al., *Rayyan—a web and mobile app for systematic reviews.* Systematic Reviews, 2016. **5**(1): p. 210.

11. Hoy, D., et al., *Assessing risk of bias in prevalence studies: modification of an existing tool and evidence of interrater agreement.* Journal of Clinical Epidemiology, 2012. **65**(9): p. 934-939.
